# Peer Review of “Levels and Predictors of Knowledge, Attitudes, and Practices Regarding Contraception Among Female TV Studies Undergraduates in Nigeria: Cross-Sectional Study”

**DOI:** 10.2196/72949

**Published:** 2025-05-08

**Authors:** Kamal Kanti Biswas

**Affiliations:** 1 IPAS Bangladesh, House 428/A, Road 30 (3rd Floor), Dhaka, Bangladesh, 880 1755592617

**Keywords:** knowledge, attitudes, practice, contraception, regression, cross-sectional, females, students, Nigeria

*This is a peer-review report for “Levels and Predictors of Knowledge, Attitudes, and Practices Regarding Contraception Among Female TV Studies Undergraduates in Nigeria: Cross-Sectional Study.”*

## Round 1 Review

### General Comments

Dear Authors,

Thank you very much for undertaking the study [1] titled “Levels and predictors of knowledge, attitude and practice of contraception among female TV undergraduates in Nigeria: a cross-sectional study” and submitting the manuscript to JMIR. The study findings are important for family planning program implementation targeting young students. I have the following comments and observations for improving your manuscript for consideration of publishing.

### Specific Comments

#### Major Comments

Introduction: line 50: “youth”: Indicate age group.

Line 52: “Utilization is higher”: Not clear what the utilization was for.

Study population: limitation: gender biased. Male involvement and attitude are equally important regarding sexually transmitted infections, particularly for male methods like use of condoms. This needs to be mentioned as a limitation of the study.

Tables all: Hastily, one sentence is used for describing findings in a table. Need to elaborate more. Further comments below.

Table 1: Rephrase the “Marital status” indicator; the data does not give the status of marriage!

Table 2: Indicate what is meant by poor, good, etc, knowledge/attitude; cite measurement scale here.

Table 3: Need to mention if this was an open-ended or structured question.

Table 4: Cite the indicators used for measuring attitude toward use of contraception.

Table 5: The predictor of not engaging in sex may be reflected well in statistical analysis, but what is the significance in real life? Why would those who had never engaged in sex have used contraception?

Discussion: Mention the rate of use of emergency contraceptive pills (ECPs) also. This is increasing in many societies. Policy makers/planners are often not aware of the need for ECPs to include a supply of ECPs in a program. A recommendation like “There may be a need to use social marketing 42 approaches to make these contraceptives available to young people to bypass the stigma they experienced while accessing 43 contraceptives from traditional sources of contraceptives” is not supported by any finding or data of the study. Rather this raises a question of bias on jumping to a solution through a particular channel. Let the program planners find out the way to resolve the issue of information availability.

Highlights: Move the highlights to the Discussion section because this is a summary of the findings.

Conclusion: Rewrite the conclusion, elaborating on recommendations per the results of the study.

## Abbreviations

ECP: emergency contraceptive pill
